# A Novel Approach of Sea Urchin-like Fe-Doped Co_3_O_4_ Microspheres for Li-S Battery Enables High Energy Density and Long-Lasting

**DOI:** 10.3390/nano13101612

**Published:** 2023-05-11

**Authors:** Nannan Yan, Xuan Zhuang, Hua Zhang, Han Lu

**Affiliations:** 1 Brain-Inspired AI Institute, Fudan University, Shanghai 200437, China; 2 State Grid East China Electric Power Test and Research Institute, Shanghai 200437, China; 3 State Grid Shanghai Electric Power Company, Shanghai 200010, China; 4 International Academy of Optoelectronics at Zhaoqing, South China Normal University, Zhaoqing 526238, China

**Keywords:** Co_3_O_4_, Fe atoms, sea urchinlike, lithium–sulfur battery

## Abstract

The poor cycle stability caused by the shuttle effect of polysulfides which have been key scientific issue in the development of high-efficiency lithium–sulfur (Li–S) batteries. In this work, the authors report a Fe-doped Co_3_O_4_ (named FCO) that was used as a sulfur-loaded host material for Li–S batteries. We demonstrate the important roles of well-designed Co_3_O_4_ particles and Fe atoms in regulating polysulfide conversion due to the strong adsorption of polysulfides by polar Co_3_O_4_, whereas Fe atoms and Co_3_O_4_ catalyze polysulfide conversion. Therefore, the LiS batteries with FCO-180 (When the hydrothermal temperature is 180 °C) sea urchinlike composites exhibited a high superior energy density (992.7 mAh g^−1^ at 0.2 C, after 100 cycles) and long-term cyclability (649.4 mAh g^−1^ at 1 C, 300 cycles) with high sulfur loading (75 wt%). This work confirms that the FCO-180 sea urchinlike increases not only the capacity of high-rate but also a generic and feasible strategy to construct practical Li–S batteries for emerging energy-storage applications.

## 1. Introduction

For largescale energy storage applications, such as new energy vehicles and energy storage systems, lithium–sulfur (Li–S) batteries have been attractive due to their ultrahigh theoretical energy density (2600 Wh kg^−1^) [1]. In addition, the elemental sulfur delivers a high theoretical capacity (1675 mAh g^−1^) based on the redox reaction, which is 3–5 folds higher than those for state-of-the-art lithium-ion batteries [2]. Meanwhile, sulfur, the main cathode material in Li–S batteries, is cheap and, with low toxicity, naturally abundant [3]. However, the commercialization of Li–S batteries has been impeded due to a series of challenges, for example, the low electronic conductivity of sulfur [4], the dissolution of high-order Lithium polysulfides (LiPSs) (Li_2_S_n_, 4 ≤ *n* ≤ 8) into the electrolyte and the shuttling of these dissolved species between the cathode and anode [5], the large volumetric expansion (≈80%) upon the conversion of S to Li_2_S [6]. These issues inevitably bring about reduced active sulfur utilization, low coulombic efficiency, unsatisfactory capacity, and poor rate performance for Li–S batteries [7].

Over the past decade, efforts have been made to design novel cathode materials to deal with these problems [8]. Among them, the excellent design of cathode materials is urgently needed for immobilizing LiPSs and enhancing the electrical conductivity of the cathode. Embedding sulfur into conductive carbon-based materials [9], such as carbon nanofibers [10], porous hollow carbons [11], and graphene [12], has been employed as a popular strategy. Although physical adsorption occurs between carbonaceous materials and LiPSs, the interaction between nonpolar carbon-based and LiPSs intermediate is weak [13]. Therefore, it is necessary to introduce polar materials into the sulfur electrode to fix the soluble LiPSs on the cathode side [14]. 

Transition metal oxides (TMOs, such as Fe_2_O_3_, MnO_2_, and Co_3_O_4_) [15,16,17] were observed to have stronger chemical bonding and catalytic effect towards LiPSs. Among them, Zhong et al. [18] found that a large amount of Li–O bounding on cobalt oxide-adsorbed LiPSs. Meanwhile, strong metal-sulfur binding was observed when adsorbing LiPSs on Co_3_O_4_, and the cyclability of the battery was greatly improved. 

Although the effective chemical adsorption of LiPSs, the low conductivity of such metal oxide material limits their application in high-performance Li–S batteries [19]. In view of this, metal materials with catalytic conversion effects have been combined with oxides to construct bifunctional compounds to improve the conversion rate of LiPSs [20]. In the work of Zhang et al. [21], they demonstrated that highly conductive Fe, Fe_3_C, and graphitic heteroatoms-doped carbon with approved electron/ion conductivity mitigated LiPS migration and regulated Li_2_S deposition/decomposition. Therefore, in order to achieve the high performance of Li–S batteries, researchers have proposed the development of a composite polar additive for sulfur cathodes [22,23,24].

Inspired by this, we present prepared sea urchinlike Co_3_O_4_ with iron (Fe) doping via facile hydrothermal and heat treatment methods. As the Fe–decorated sea urchinlike materials evidently improved electron conductivity. Benefiting from polar Fe and Co_3_O_4_, which provide strong chemical adsorption on LiPSs. The sea urchinlike structure and Fe atoms with large specific surface area provide enough reaction sites for electrochemical reaction, delivering the discharge capacity was 774.9 mAh g^−1^ when the cells tested the rate capability at 2 C. This unique sea urchinlike Fe/C_3_O_4_ cathode offered stable discharge capacity over 300 cycles at 1 C with a coulombic efficiency close to 100%, opening up a new thought to obtain an excellent sulfur host in high-performance Li–S batteries.

## 2. Experimental Section

### 2.1. Preparation of FCO

Firstly, 3 g of glucose was added in 30 mL of DI water, stirring for 30 min, then 5.24 g cobalt nitrate hexahydrate (Co(NO_3_)_2_·6H_2_O) and 0.73 g iron nitrate nonahydrate (Fe(NO_3_)_3_·9H_2_O) were added in it, stirring at 450 rpm to obtain a transparent pink solution.

After stirring for 60 min, the mixture was added into a 150 mL Teflon-line autoclave and heated at 160 °C, 180 °C, and 200 °C for 400 min. After the hydrothermal reaction, coffee-colored precipitate materials were obtained. The products were separated by centrifugation and washed with deionized water four times, with the product finally dried at 60 °C under vacuum.

Finally, the above samples were obtained, and then they were heated for 1 h at 500 °C in a muffle furnace. The FCO materials with different morphologies were gathered after cooling to room temperature.

### 2.2. Synthesis of S@FCOx Cathode 

In a typical strategy, FCOx was mixed with commonly commercial sulfur with a weight ratio of 3:1 by milling to be active material. Whereafter, the mixture was placed into a 50 mL Teflon bottle, followed by a seal with argon gas, then heated at 155 °C for 12 h to obtain S@FCOx.

### 2.3. Physicochemical Characterization

The micro-morphologies of the FCO-160, FCO-180, and FCO-200 materials were characterized by SEM (Hitachi, Hitachi S4800, Tokyo, Japan) and TEM (HRTEM) (JEOL, JEM 2100, Tokyo, Japan). The Energy Dispersive Spectrometer (EDS) mapping of FCO-180 was also characterized. The crystalline structures of the FCO-160, FCO-180, and FCO-200 samples were acquired on an Xray diffractometer (XRD, Rigaku, Rigaku-TTRIII, Austin, TX, USA) within an angular range of 10–80°. The superficial chemical compositions were determined by Xray photoelectron spectroscopy (XPS, Thermo Scientific K-Alpha, Waltham, MA, USA). TGA (TG/DTA 6300, NSK, Tokyo, Japan) was collected with a TGA thermogravimetric analyzer under nitrogen gas (N_2_) at a temperature range from room temperature to 500 °C. The VSorb X800 tester based on N_2_ adsorption–desorption isotherms recorded Brunauer–Emmett–Teller (BET) specific surface area of the FCO-160, FCO-180, and FCO-200 samples. 

### 2.4. Electrochemical Measurements

Electrochemical lithium storage performance and rate capability were evaluated in a CR2032 coin-type cell with FCO as a working electrode. A conventional sulfur cathode (S@FCO composite = 3/1 (*w*/*w*))/polyvinylidene fluoride (PVDF) binder/acetylene black = 70/10/20 (*w*/*w*/*w*) on an Al foil current collector. Lithium metal as an anode, microporous membrane (Celgard^®^2500) as a separator, and electrolyte (1 M LiTFSI in a mixed solution of 1,2-dimethoxy ethane (DME) and 1,3dioxolane (DOL) (1:1 *v*/*v*)). CR2032 button cells were assembled in the glove box. The galvanostatic charging and discharging measurements were performed in a potential range from 1.7 V to 2.8 V to measure the rate capability and cycling stability of the batteries by using the NEWARE battery tester. Specific capacity values were calculated based on the content of the sulfur element. The electrochemical impedance spectroscopy (EIS) was performed using an electrochemical workstation (CHI 660) over the 0.01–100 kHz frequency range. Then, cyclic voltammetry (CV) tests were also measured with the electrochemical workstation from 1.7 to 2.8 V. The UV–vis spectra were determined at 200–600 nm spectral range on a UV spectrophotometer (UV1800PC).

## 3. Results and Discussion 

**Figure 1:**
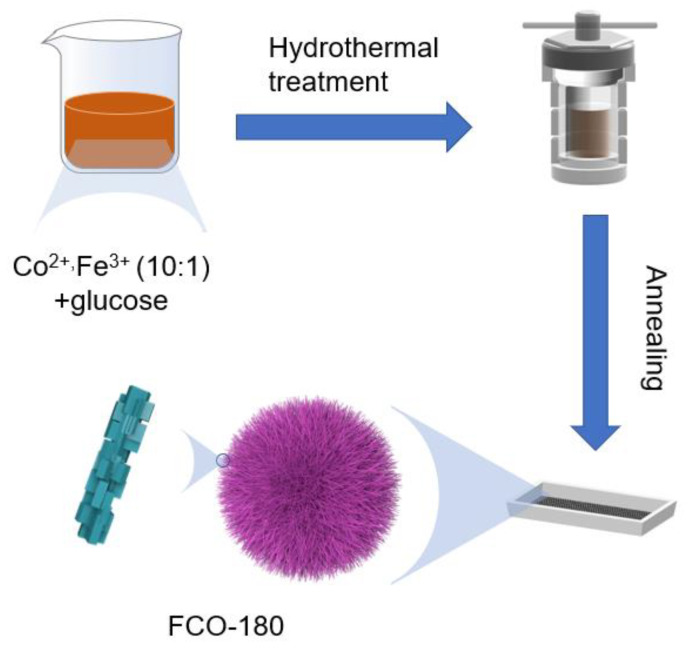
Schematic illustration of the synthesis process for FCO-180.

**Figure 2:**
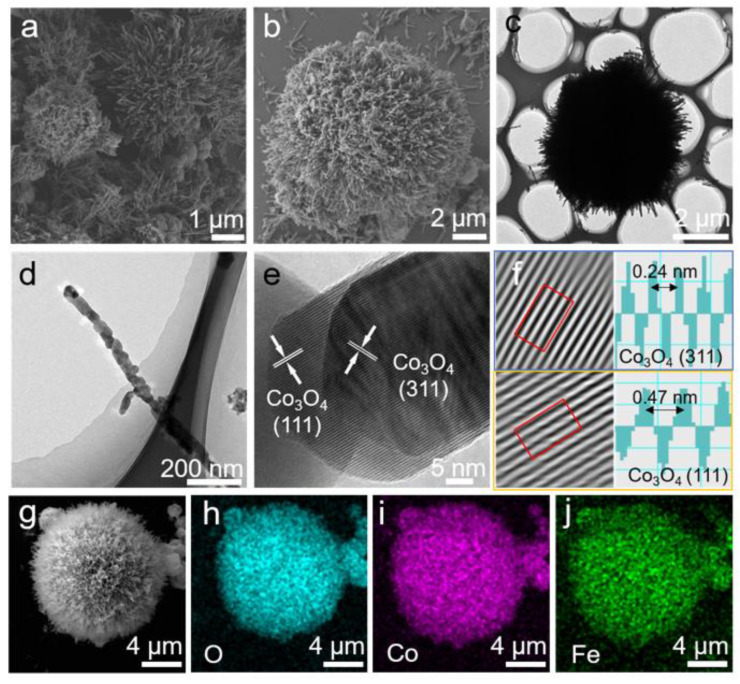
(**a**,**b**) SEM images of FCO-180; (**c**,**d**) TEM image of ultrathin FCO-180 and detailed structures; (**e**,**f**) the HRTEM of FCO-180; (**g**–**j**) elemental mapping of FCO-180.

The preparation route of FCO-180 was detailly illustrated in Figure 1. FCO with different temperatures of hydrothermal reaction was prepared as a reference. Observing from SEM images (Figure 2a,b and Figure S1), the sea urchinlike structure was well maintained only for FCO-180. FCO-180 presents regular sphericity with a uniform diameter of 5–13 μm. This morphology was also detected from the TEM (Figure 2c). Moreover, the shape of the FCO-180 sample is sea urchinlike, which could provide more of the specific surface area. In Figure 2d, each Fe atom has a size of about 20–30 nm, and Fe atoms are dispersed homogeneously in Co_3_O_4_. As shown in Figure 2e,f (the high-resolution (HR)TEM), there are two evident crystal lattice fringe distances indexed to the (311) and (111) plane of Co_3_O_4_, respectively. The EDS mapping of FCO-180 spheres further confirmed the uniform distribution of O, Co, and Fe (Figure 2g–j) in the formed sea urchinlike structure.

**Figure 3:**
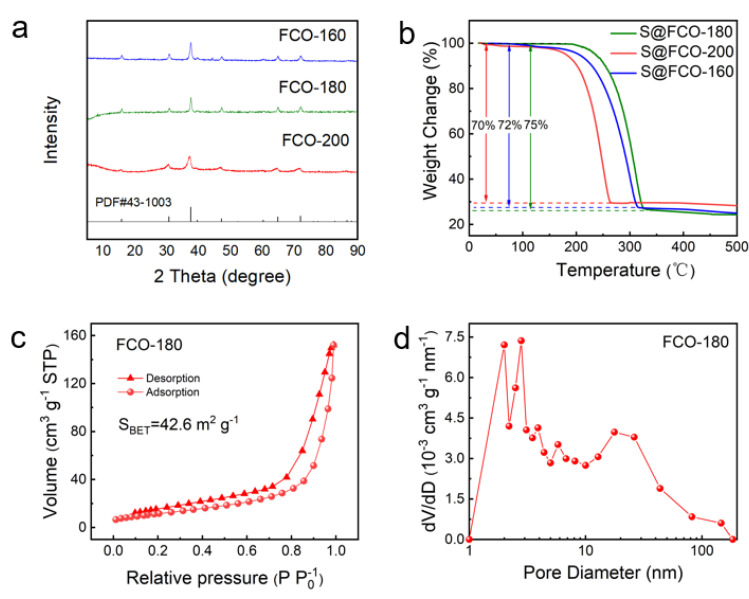
(**a**) XRD pattern of FCO-180, FCO-160, and FCO-200; (**b**) The TGA curves of S@FCO-180, S@FCO-160, and S@FCO-200 under N_2_ atmosphere; (**c**) The Nitrogen adsorption–desorption isotherms of FCO-180; (**d**) pore size distribution of FCO-180.

For comparison, samples dried at 160 °C and 200 °C were prepared and named FCO-160 and FCO-200, respectively. In Figure 3a, there are Xray diffraction (XRD) patterns of FCO-160, FCO-180, and FCO-200. The FCO-180 exhibits diffraction peaks at 18.9, 31.4, 36.6, and 65.28° that can be ascribed to the (111), (220), (311), and (440) planes of Co_3_O_4_ (PDF#431003). Moreover, the signals of iron nanoparticles and their oxides were not observed in the diffraction peaks of FCO-160, FCO-180, and FCO-200, further indicating that the highly dispersed Fe atoms were successfully doped within Co_3_O_4_. Additionally, XRD data around the main FCO peak at around 36.6° were enlarged in Figure S2. It is interesting that the XRD diffraction peaks around 36.6° are shifted to small angles with increasing annealing temperature. The reason for this phenomenon may be due to the increase in temperature, which leads to the increase of lattice oxygen defects within the oxide system, thus causing lattice distortion. As seen in Figure 3b, the thermogravimetric curve reveals that the S content in the S@FCO-180 sea urchinlike is about 75 wt%. The higher sulfur content in the S@FCO-180 than that of S@FCO-160 and S@FCO-200, would promise a practical application for sulfur cathode material and prove the sulfur loading ability of the FCO-180 sea urchinlike structure.

As shown in Figure 3c,d and Figure S3, the N_2_ adsorption–desorption isotherms were applied to study the specific surface area and pore structure of FCO-180, together with FCO-160 and FCO-200 for comparison. In Figure 3c, the FCO-160, FCO-180, and FCO-200 samples show typical type-Ⅲ nitrogen adsorption–desorption isotherms with a clear H_3_ hysteresis loop, indicating the presence of mesopores. The specific surface area of FCO-180 is 42.6 m^2^ g^−1^ in Figure 3c, which is much higher than that of 39.1 m^2^ g^−1^ and 31.6 m^2^ g^−1^ of FCO-160 and FCO-200, respectively. This high surface area is mainly attributed to the sea urchinlike structure and the accumulation of Fe atoms, just as observed in the TEM image (Figure 1c,d). The size of mesopores was measured to be 2–17 nm, with an average pore size of 13.8 nm. It not only benefits the penetration of electrolytes but also provides effective pore space for the increase of sulfur content (Figure 3d).

**Figure 4:**
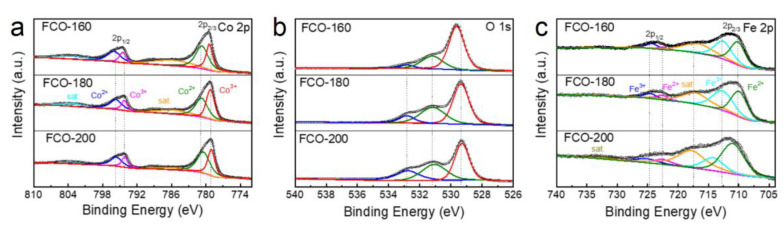
(**a**) High-resolution XPS spectra of Co 2p in the FCO-160, FCO-180, and FCO-200; (**b**) O 1s in the FCO-160, FCO-180, and FCO-200; (**c**) Fe 2p in the FCO-160, FCO-180, and FCO-200, respectively.

In Figure 4, X-ray photoelectron spectroscopy (XPS) was presented to evaluate the composition and nature of bonding of the elements in the FCO-160, FCO-180, and FCO-200 samples, respectively, where Co 2p (Figure 4a), O 1s (Figure 4b) and Fe 2p (Figure 4c) could be observed. The Co 2p spectrum of FCO-180 samples shows a doublet consisting of spaced spinor–bit components from 2p_3/2_ and 2p_1/2_. The peaks of Co^2+^ 2p_3/2_ and Co^2+^ 2p_1/2_ are located at 780.6 and 795.7 eV, respectively, and the binding energies at approximately 779.3 and 794.3 eV are characteristic peaks of the Co^3+^ state. This is further verified by the peak in Figure 4a, which is typical for Co_3_O_4_ particles [25]. Figure 4b shows the high-resolution XPS spectra of O 1s; the curve can be divided into three, which are centered at 532.8, 531.2, and 529.4 eV, corresponding to the signals of adsorbed O, defective O, and lattice O, respectively [17]. Three characteristic peaks of O 1s shift when temperature changes (Figure 4b). Demonstrated by the Fe 2p spectrum (Figure 4c), the Fe 2p_1/2_ orbital response bands at 723.7 eV and 2p_3/2_ orbital response bands at 710.6 eV, suggesting the successful introduction of Fe atoms into the sea urchinlike structure. 

**Figure 5:**
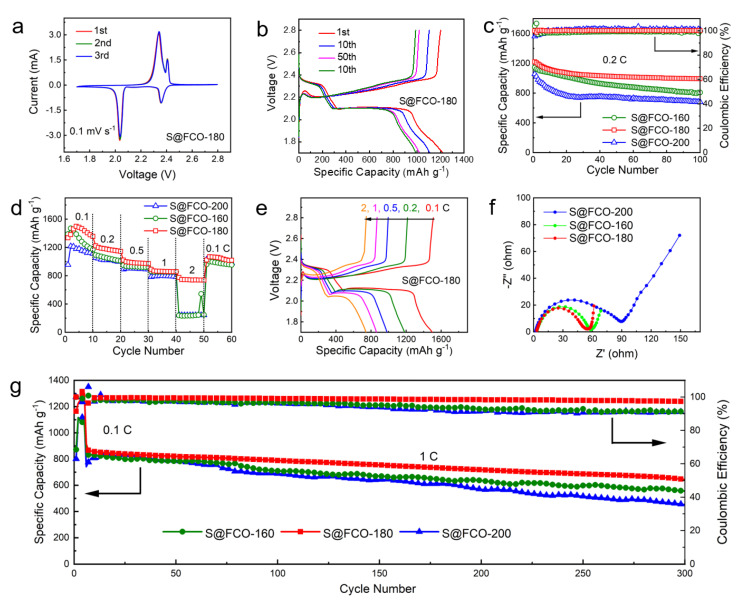
(**a**) The first three CV curves of S@FCO-180; (**b**) Charge/discharge profiles of S@FCO-180 at a rate of 0.2 C; (**c**) The cycling performance of S@FCO-180 at a rate of 0.2 C; (**d**) Rate capability of S@FCO-160, S@FCO-180, and S@FCO-200; (**e**) Charge/discharge profiles with S@FCO-180 at different current densities; EIS spectra of S@FCO-160, S@FCO-180, and S@FCO-200 (**f**); (**g**) The cycling performance of S@FCO-160, S@FCO-180, and S@FCO-200 at 1 C.

The cycle voltammetry (CV) curves of S@FCO-180 (Figure 5a) revealed a general electrochemical response at a scanning rate of 0.1 mV s^−1^. The CV plots revealed the S@FCO-180 electrode exhibited two oxidation peaks and two distinct reduction peaks, which corresponded to the redox couples and phase transformations during the overall charge/discharge process (Figure 5a,b). Two peaks were detected at 2.35 V and 2.03 V during the cathodic sweep, which symbolized the retention level of LiPSs from S_8_ to soluble Li_2_S_n_ (4 ≤ *n* ≤ 8), and finally to insoluble Li_2_S. Several oxidation peaks appeared near 2.34 V and 2.40 V during the reverse scanning process, associated with the oxidation process of Li_2_S to long-chain LiPSs, and further oxidized to S_8_. After the first cycle, the cathodic and anodic peaks curves of the second and third cycles well overlapped each other, suggesting that the S@FCO-180 electrode has good stability and reversibility. As shown in Figure S4a,b, S@FCO-160, and S@FCO-200 composites showed CV curves similar to those of S@FCO-180 composites, but the S@FCO-180 composites exhibited higher peak intensities and significantly lower polarization, indicating faster reaction kinetics and much more reversible conversion reaction.

The S@FCO-180 electrode showed well-defined charge/discharge plateaus at 0.2 C corresponding to the abovementioned CV profiles (Figure 5b). For comparison, the corresponding electrochemical measurement of S@FCO-160 and S@FCO-200 cathodes were presented in Figure S4c,d. The discharge capacity of the S@FCO-180 electrode was 1220.4 mAh g^−1^, which was higher than the S@FCO-160 and S@FCO-200 electrodes. The decay rate of the discharge capacity of the S@FCO-180 electrode from the 1st to the 100th cycle is lower than that of the S@FCO-160 and S@FCO-200 electrodes. Figure 3c reveals the cycling performance of cells with S@FCO-160, S@FCO-180, and S@FCO-200 electrodes at 0.2 C for 100 cycles. The specific capacity reached 992.8 mAh g^−1^ after 100 cycles at 0.2 C, which was about 81.4% of the initial capacity. The excellent cycling properties and the high coulombic efficiency of the S@FCO-180 cathode can be attributed to the sea urchinlike and enhanced electrical conductivity by the doping of Fe atoms.

Rate capability tests for S@FCO-160, S@FCO-180, and S@FCO-200 electrodes were conducted, as shown in Figure 5d. Among the three, S@FCO-180 showed higher capacity values. The discharged capacities of the S@FCO-180 cathode were 1498.0, 1222.2, 1020.4, 890.1, and 774.9 mAh g^−1^, which maintained at the increased current densities of 0.1, 0.2, 0.5, 1.0 and 2.0 C, respectively. When the current density was increased to 2 C, the discharge capacity still maintained up to 774.9 mA h g^−1^, showing about 51.7% of the first capacity. In comparison to the S@FCO-180 cell, the capacity retained rate at 17.2% and 20.2% of S@FCO-160 and S@FCO-200 after experiencing this series of current density changes, showing an inferior electro/ion transfer and polarization of LiS batteries with different current densities. Even when the current density was switched back to 0.1 C, the discharge capacity of the S@FCO-180 cell recovered to 1022.1 mAh g^−1^. The dramatic capacity decline of the S@FCO-160 and S@FCO-200 cathode can be attributed to the severe shuttle effect. Additionally, the charge/discharge curves still showed typical voltage plateaus and delivered a high capacity at a high current density at 2 C, indicating that S@FCO-180 has a good rate performance (Figure 5e and Figure S4e,f).

To explore the merit of designed S@FCO-160, S@FCO-180, and S@FCO-200 electrodes, EIS was used, as shown in Figure 5f. As illustrated in Figure 5f, the S@FCO-180 electrode showed a low resistance of 54 Ω, whereas a higher resistance of 58 Ω and 90 Ω was found in the S@FCO-160 and S@FCO-200 electrodes before cycling. The Nyquist plot displayed a semicircle followed by a straight line corresponding to the charge transfer resistance (Rct) and Warburg resistance (Z_W_), respectively. As shown in Figure 5f, among three curves, the S@FCO-180 electrode showed a smaller charge transfer resistance and a faster charge transfer between S@FCO-180 and LiPSs. The R_ct_ significantly decreased for the S@FCO-180 electrode as compared to S@FCO-160 and S@FCO-200 electrodes, which might be mainly because of the introduction of more Fe/Co_3_O_4_ nanoparticles into the sea urchinlike structure. The Fe/Co_3_O_4_ nanoparticles and sea urchinlike structure can offer more sufficient pathways for electrons to reach FCO-180/LiPSs interface and bring more active sites inside the reaction interface.

Then Figure 5g displayed the long-cycle performance of LiS batteries, which were tested at 1 C after activating at 0.1 C for two cycles. The initial discharge capacity of the cell with S@FCO-180 was about 867.0 mA h g^−1^. In addition, the average capacity fading rate was as low as 0.085% per cycle for 300 cycles, much better than the cells with other electrodes, including S@FCO-160 and S@FCO-200 electrodes. The excellent results of S@FCO-180 proved that complete encapsulation of sulfur inside Fe/Co_3_O_4_ sea urchinlike is an effective way of achieving high cycle stability.

**Figure 6:**
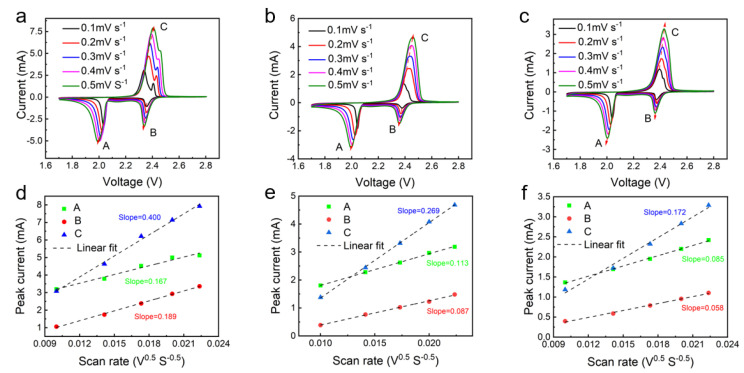
CV curves of FCO-180 (**a**), FCO-160 (**c**), and FCO-200 (**e**) electrodes at different scan rates ranging from 0.1 to 0.5 mV s^−1^ and corresponding linear relationships (**b**,**d**,**f**) between CV curves peak currents versus square root of the voltage scan rate.

To further explore the diffusion process of lithium ions, cyclic voltammetry is devoted to investigating the conversion kinetics of the LiPSs in the LiS batteries. The diffusion characteristics of Li^+^ of FCO-160, FCO-180, and FCO-200 electrodes were investigated by CV analysis at scan rates ranging from 0.1 to 0.5 mV S^−1^ in Figure 6. All LiS cells presented two well-separated reduction peaks, which corresponded to the reduction of elemental S to Li_2_S via intermediate LiPSs. In addition, the peak positions showed a slight shift with the scan rate increasing. Additionally, the oxidation peak was related to the reverse process, that is, the oxidation of Li_2_S/Li_2_S_2_ to Li_2_S_x_ (4 < x < 8) until Li_2_S_x_ (4 < x < 8) was completely oxidized to elemental sulfur, thus completing the redox cycle. The lithium-ion diffusion properties were obtained by using the Randles–Sevcik equation:

I_p_ = (2.69 × 10^5^) *n*^1.5^ AD_Li+_^0.5^ + ∆C_Li+_ + ν^0.5^

In this formula, Ip represents the peak current, *n* is the number of electrons transferred by charge (in LiS batteries, *n* = 2), A is the cathode area, D_Li+_ represents the diffusion coefficient of lithium ions, and C_Li+_ stands for the concentration of Li ions, and ν represents the scan rate of cyclic voltammetry. Here I_p_ and ν^0.5^ are the only two variables; the diffusion coefficient of Li^+^ (D_Li+_) can be directly reflected by the slope of the fitting curve (Ip/ν^0.5^) [26,27,28]. Based on the RandlesSevcik equation, the current of the peak with S@FCO-180 electrode is higher at the same scanning speed, and the higher slope is obtained from the curves fitting process. The above result suggests that the presence of FCO-180 sea urchinlike can promote the rapid transfer of Li^+^, which can be attributed to the fast ion exchange and redox kinetics of FCO-180 sea urchinlike materials.

**Figure 7:**
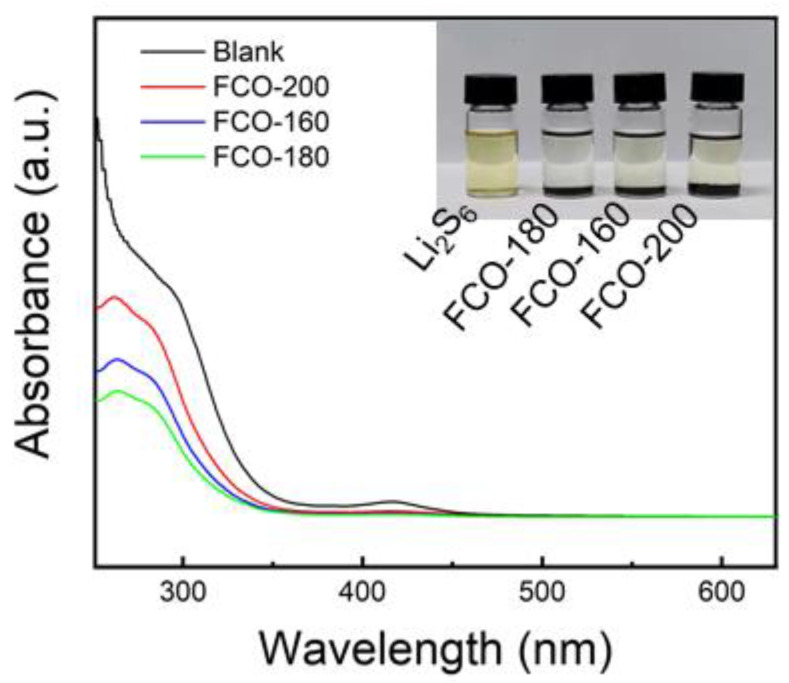
LiPS’s adsorption tests for FCO-160, FCO-180, and FCO-200, respectively, and the optical photo of the visible adsorption experiment.

The visualized adsorption ability test of LiPSs and UVvis spectroscopy was conducted to further probe the chemical interaction between different cathode materials (S@FCO-160, S@FCO-180, and S@FCO-200) and LiPSs, using Li_2_S_6_ as the representative LiPSs. As displayed in Figure 7, the S@FCO-180 samples completely decolored the Li_2_S_6_ solution, whereas the Li_2_S_6_ solution containing S@FCO-160 and S@FCO-200 turned colors from yellow to light yellow. In addition, the UV–Vis spectra showed that peaks of Li_2_S_6_ were significantly weaker at 262.0 nm when S@FCO-180 hosts were added to the solution. The strong chemisorption between FCO-180 and LiPSs resulted in decreased peak intensities during the tests. These results further confirm the good LiPS adsorption ability of the FCO-180 samples. 

**Figure 8:**
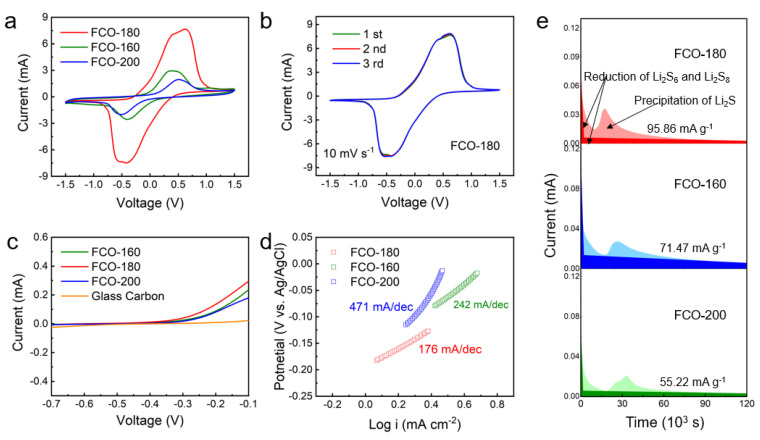
(**a**) CV curves of FCO-160, FCO-180, and FCO-200 symmetric batteries; The first three CV cycles of the FCO-180 (**b**) symmetric cell; (**c**) LSV curves and the corresponding (**d**) Tafel plots of different electrodes at 8 mV s^−1^; (**e**) Potentiostatic discharge curves employing FCO-160, FCO-180, and FCO-200 as electrode at 2.05 V.

The CV profiles of the symmetric cells with FCO-160, FCO-180, and FCO-200 as the counter electrodes are in Figure 8a. The symmetric cells were realized using FCO-160, FCO-180, and FCO-200 as both working/counter electrodes at a scan rate of 10 mV s^−1^ and 0.2 mol L^−1^ Li_2_S_6_ solution as the electrolyte. It was clear that the FCO-180 electrode exhibited a stronger peak intensity and smaller redox polarization from CV curves, compared with the FCO-160 and FCO-200 electrodes, suggesting that the FCO-180 composites played relatively good electrocatalytic activity and accelerated conversion of LiPSs [29,30]. The excellent stability of FCO-180 as an efficient electrocatalyst redox reactions of LiPSs could be confirmed by the well-overlapping peaks of CV profiles in the Figure 8b. It is related to the fact that the Fe atoms are uniformly dispersed in the FCO-180 sea urchinlike, and the FCO-180 has a larger effective surface and more active sites. 

Furthermore, the reaction kinetics of the cells are evaluated based on a linear sweep voltammetry (LSV) measurement. As shown in Figure 8c, the FCO-180 sea urchinlike enabled a more positive onset potential and higher current response for Li_2_S oxidization compared with those of FCO-160 and FCO-200. Clearly, FCO-180 sea urchinlike presented lower Tafel slopes than FCO-160 and FCO-200 for both the lithiation and delithiation processes in Figure 8d, suggesting that the conversion from short-chain Li_2_S to long-chain LiPSs was easier and good catalytic performance [31,32].

In general, the amount of nucleation and decomposition of Li_2_S during the redox reaction determines the specific capacity of the LiS batteries. In Figure 8e, the nucleation and growth of Li_2_S and the reduction of Li_2_S_8_ and Li_2_S_6_ corresponded to different color shades. The experiments showed that the FCO-160 and FCO-200 electrodes exhibited much later and lower current peaks than the FCO-180 electrode. In addition, the capacity of Li_2_S precipitation on FCO-180 (95.86 mAh g^−1^) was higher than that on FCO-160 (71.47 mAh g^−1^) and FCO-200 (55.22 mAh g^−1^). According to the results, the FCO-180 can act as a catalyst to accelerate the conversion of liquid LiPSs to solid Li_2_S.

## 4. Conclusions

In summary, we successfully prepared a sulfur-loaded host material for Li–S batteries, FCO-180 sea urchinlike, where Fe atoms were decorated on Co_3_O_4_. The cell with an FCO-180 electrode exhibits excellent electrochemical performance, and the improved stability is mainly attributed to the Fe/Co_3_O_4_ and sea urchinlike structure. The shuttle effect is effectively alleviated by the combination of sea urchinlike structure and chemisorption of FCO-180. Thus, the S@FCO-180 shows high specific capacity, excellent rate capability, and a capacity decay of 0.085% per cycle over 300 cycles at 1 C. Our all-purpose design strategy, bridges the conventional TMOs and promising metal atoms together, pioneering a new and viable avenue for the development of advanced Li–S batteries.

## Data Availability

Not applicable.

## Supplementary Materials

The following supporting information can be downloaded at: https://www.mdpi.com/article/10.3390/nano13101612/s1, Figure S1: SEM image of FCO-160 composite and FCO-200 composite; Figure S2: Enlarged XRD patterns of FCO-180, FCO-160 and FCO-200; Figure S3: The Nitrogen adsorption−desorption isotherms and pore size distribution of FCO-160 and FCO-200; Figure S4: CV curves of S@FCO-160 (a) and S@FCO-200 (b) electrodes in the potential range of 1.7–2.8 V at 0.1 mV s^−1^; The discharge/charge voltage profiles of (b) S@FCO-160 and (e) S@FeCO-180 electrodes at 0.2C; Reversible discharge/charge voltage profiles of (c) S@FCO-160 and (f) S@FeCO-180 electrodes at different current densities (from 0.1 to 2 C) .
