# Supplementary material for: A Novel Approach of Sea Urchin-like Fe-Doped Co3O4 Microspheres for Li-S Battery Enables High Energy Density and Long-Lasting
Source: Nanomaterials (Basel). 2023 May 11;13(10):1612. doi: 10.3390/nano13101612 (PMC10220560; doi:10.3390/nano13101612)
Supplement: Supplementary file 1 [file nanomaterials-13-01612-s001.zip › nanomaterials-2324134-supplementary.pdf]

Supplementary Materials

A Novel Approach of Sea Urchin-like Fe-Doped Co_3O_4 Microspheres for Li-S Battery Enables High Energy Density and Long-Lasting

Nannan Yan [1,2], Xuan Zhuang [3], Hua Zhang [4] and Han Lu [4,*]

1 Brain-inspired AI Institute, Fudan University, Shanghai 200437, China
2 State Grid East China Electric Power Test and Research Institute, Shanghai 200437, China
3 State Grid Shanghai Electric Power Company, Shanghai 200010, China
4 International Academy of Optoelectronics at Zhaoqing, South China Normal University, Zhaoqing 526238, China
* Correspondence: han.lu@zq-scnu.org

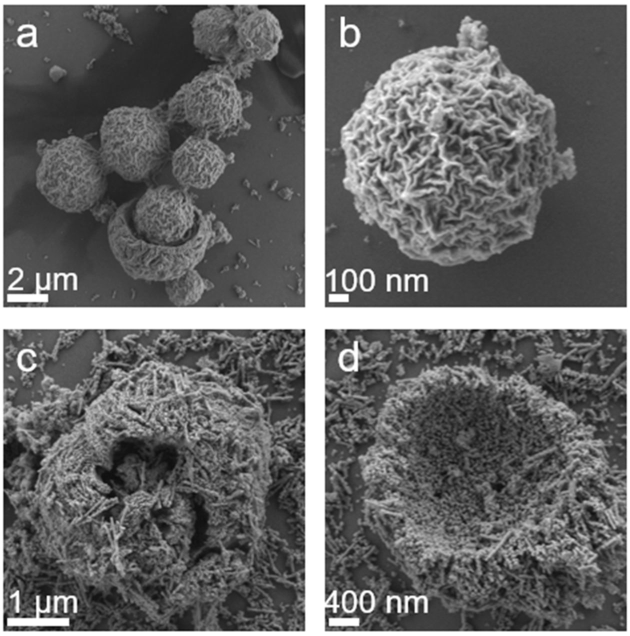

Figure S1. SEM image of FCO-160 composite (a, b) and FCO-200 composite (c, d).

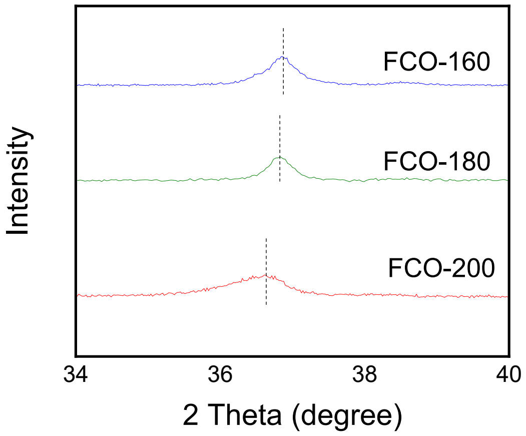

Figure S2. Enlarged XRD patterns of FCO-180, FCO-160 and FCO-200.

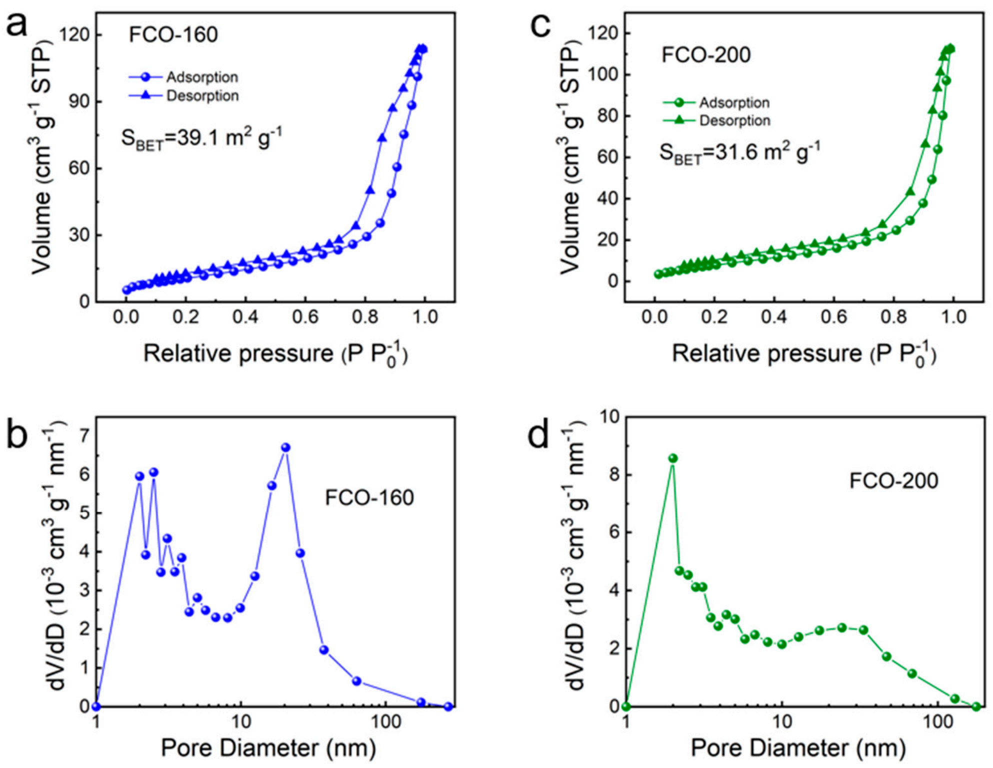

Figure S3. (**a**,**c**) The Nitrogen adsorption-desorption isotherms of FCO-160 and FCO-200; (**b**,**d**) pore size distribution of FCO-160 and FCO-200.

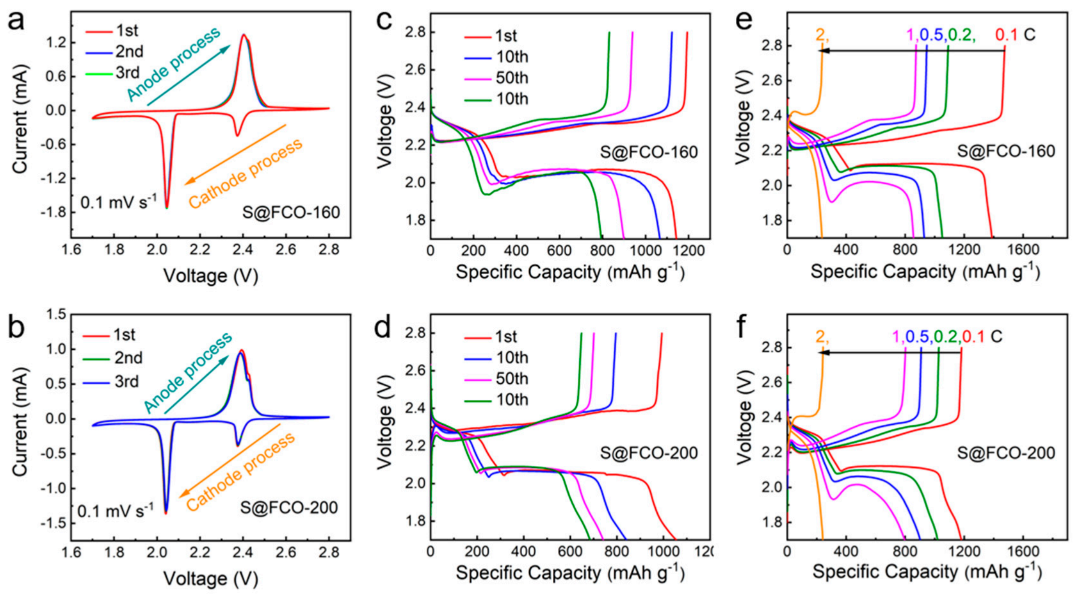

Figure S4. CV curves of S@FCO-160 (**a**) and S@FCO-200 (**b**) electrodes in the potential range of 1.7–2.8 V at 0.1 mV s^{-1}; The discharge/charge voltage profiles of (**b**) S@FCO-160 and (**e**) S@FeCO-180 electrodes at 0.2 C; Reversible discharge/charge voltage profiles of (**c**) S@FCO-160 and (**f**) S@FeCO-180 electrodes at different current densities (from 0.1 to 2 C).
